# Is the coupling and coordination of economic, social and environmental development crucial to the governance of relative poverty?

**DOI:** 10.1371/journal.pone.0306641

**Published:** 2024-08-02

**Authors:** Wei Huang, Shuhui Gao, Peiqi Hu, Yue Han, Shiyu Ding

**Affiliations:** Department of Management and Economics, North China University of Water Resources and Electric Power, Zhengzhou, Henan, China; Pontifical Catholic University of Rio de Janeiro: Pontificia Universidade Catolica do Rio de Janeiro, BRAZIL

## Abstract

As the primary goal of the 17 Sustainable Development Goals (SDGs), poverty eradication is still one of the major challenges faced by countries around the world, and relative poverty is a comprehensive poverty pattern triggered by the superposition of economic, social, and environmental dimensions. Therefore, Therefore, this paper introduces the perspective of coupled coordination to consider the formation of relative poverty, constructs indicators in three major dimensions: economic, social, and environmental, proposes a fast and more accurate method of identifying relative poverty in a region by using machine learning, measures the degree of coupled coordination of China’s relatively poor provinces using a coupled coordination model and analyzes the relationship with the level of relative poverty, and puts forward suggestions for poverty management on this basis using typology classification. The results of the study show that: 1) the fusion of data crawlers, remote sensing space, and other multi-source data to construct the dataset and propose a fast and efficient regional relative poverty identification method based on big data with low comprehensive cost and high identification accuracy of 0.914. 2) Currently, 70.83% of the economic-social-environmental systems of the relatively poor regions are in the dysfunctional type and are in a state of disordered development and malignant constraints. The regions showing coupling disorders are mainly clustered in the three southern prefectures of Xinjiang, Qinghai, Gansu, Yunnan, and Sichuan, and their spatial distribution is relatively concentrated. 3) The types of poverty and their coupled and coordinated development in each region show large spatial variability, requiring differentiated poverty eradication countermeasures tailored to local conditions to achieve sustainable regional economic-social-environmental development.

## 1. Introduction

Poverty has been a persistent and multifaceted problem throughout human history. The United Nations has made the "eradication of poverty in all its forms" the overarching goal of the 17 sustainable development goals (SDGs) [1]. The ongoing fight against poverty remains a major global challenge, especially for developing countries. As the world’s largest developing country, China had historically had a large number of rural poor [2], which had seriously impeded economic progress and social development. The Chinese Government attaches great importance to poverty alleviation and development and has implemented a series of poverty alleviation programs aimed at eradicating poverty [3]. According to current standards, by 2020, all 98.99 million rural poor will have been lifted out of poverty, all 832 poor counties will have been removed, and all 128,000 poor villages will have been added to the list; while absolute poverty has been eradicated in the short term, relative poverty remains a persistent challenge that will continue to affect long-term sustainable economic and social development [4].Therefore, alleviating the problem of relative poverty is an important guarantee for the successful realization of sustainable development goals.

Much progress has been made in poverty eradication, with the earliest poverty eradication strategies tending to focus only on economic dimensions of growth, arguing that poverty is primarily caused by inadequate economic conditions or low incomes [5–7]. However, as the understanding of poverty has deepened, more and more studies have begun to emphasize a multidimensional view of poverty, which is considered to be more constrained by resource endowments such as the environment, society, and ecology, and to be the result of a combination of factors [8, 9]. There is also an interactive relationship between the economy, society, and the environment. Western scholars have also verified the natural geographic factors of poverty through different types of countries in the world, and the results of the study show that the poor natural geography has led to a spatial poverty trap where the distribution of the poor population is concentrated in various countries, which has a serious negative impact on economic development [10]; the impact of the "geographic capital" factor on the growth of income of the rural poor households is significant, mainly in the social development of the backwardness and geographic location disadvantage [11]. Coupling of the economic, social, and environmental dimensions as a key pathway to sustainable poverty eradication [12, 13].

In an attempt to address the issue of relative poverty through the coupling of the economic, social, and environmental dimensions to achieve sustainable development, the following issues must be addressed. 1)In the current period, what are the main areas of relative poverty in China? What is their poverty situation? 2) What is the cur rent relationship between subsystems within the poverty area and are they coordinated? 3) How to accurately identify the key elements of the region that affect the realization of sustainable poverty eradication?

To solve the above problems, this paper carries out systematic quantitative research in terms of the identification of relatively poor areas, the measurement of the coupling coordination level, and the analysis of poverty alleviation paths, etc. The results of the research are as follows: 1) Through the fusion of multi-source data such as data crawler, remote sensing space, and other sources of data to construct the dataset, it puts forward a fast and efficient method of identifying the relative poverty of a region based on big data, with a low comprehensive cost and a high identification accuracy of 0.914. (2) Currently, 70.83% of the relatively poor regions have their economic-social-natural systems belonging to the dysfunctional type, and are in a state of disordered development and vicious constraints, and this part of the region is mainly clustered in Northwest China, with a relatively centralized spatial distribution. (3) The current types of poverty in the relatively poor regions are the economic relative poverty type, the economic-social two-factor relative poverty type, the economic-natural two-factor relative poverty type, and the Economic-Social-Natural Multi-Factor Relative Poverty Type, of which the regions belonging to the Economic-Social-Environmental Multi-Factor Relative Poverty Type are the most numerous, accounting for 68.75%.

This study contributes to the literature in the following three ways.1) Compared with previous studies that only use yearbook data at the socioeconomic level, this paper integrates heterogeneous data from multiple sources, such as crawler data, statistical data, and spatial data, to cover the development status of the region at the economic, social, and natural levels, and ultimately unifies the resulting data into quarterly scales, which can comprehensively measure the overall development status of the region and improve the timeliness of identifying relatively poor regions. 2) This paper applies machine learning techniques to the study of regional relative poverty to obtain an efficient and more accurate XGBoost model. This not only comprehensively measures the overall development of the region, but also improves the timeliness of identifying relatively poor regions to a certain extent.2) This paper applies machine learning technology to the study of regional relative poverty and obtains an XGBoost model for efficiently and accurately identifying relatively poor regions.3) Introduces the perspective of coupling into the problem of relative poverty, classifies the types of coupling coordination through the coupling coordination measurement of the economy, society, and the environment, and puts forward targeted recommendations for each region. The study proposes targeted recommendations for each region. Through systematic quantitative analysis, this study contributes to alleviating the relative poverty problem and realizing regional sustainable development.

## 2. Literature review

### 2.1 Poverty and sustainable development

#### 2.1.1 Relative poverty measurement

The development of standards for measuring relative poverty is fundamental to addressing the problem of poverty identification. Despite numerous studies on this topic, there is currently no standardized approach [14]. Two primary methods for measuring relative poverty are the income proportion method and the multidimensional poverty index measurement method. 1) The income proportion method, as proposed by Townsend, involves setting the poverty line as a certain percentage of the average income [15]. Internationally, the Gini coefficient is commonly used to measure income distribution differences and reflect relative poverty. In domestic settings, various studies have suggested using a percentage of the median income of urban and rural residents as the relative poverty standard (Table 1), with adjustment cycles of 5–10 years [16]. 2) The multidimensional poverty index measurement method was advocated by Easterlin Paradox in 1974, emphasizing the need to establish a multidimensional poverty index to address the limitations of income-based measurements [17]. Amartya Sen highlighted the relative deprivation of economic and social rights as a key aspect of relative poverty [18], while the United Nations Development Program constructed the Multidimensional Poverty Index (MPI) based on health, education, and standard of living dimensions [19]. Alkire proposed the A-F methodology, which considers multiple dimensions such as income, education, health, and living conditions to measure relative poverty [20]. In conclusion, while there is no consistent standard for defining relative poverty, the methods for measuring it provide valuable guidance; The World Bank and other international organizations have come up with the SEID index, which measures poverty in terms of per capita income, employment rate, education level, sanitation facilities and so on [21]. At the same time, with the rapid development of the information field, when measuring relative poverty through the establishment of multidimensional poverty indicators, measuring and predicting through machine learning methods are gradually applied to the field of poverty [22]. Identification of the 20% of regions with the lowest level of disposable income per capita as areas of relative deprivation.

**Table 1:** Relative poverty lines by country.

Region/organization	Methodologies	Relative poverty line criteria
OECD	Relative income standard method	50% of median or average income
EU		Median disposable income per capita 60%
Japanese	Relative Equalization of Living Standards Act	Per capita living consumption expenditure of low-income households reaches 60% of that of middle-income households
United States of America	Criteria for proportionality based on total population	Lowest 10%-15% of the country’s income
United Kingdom of Great Britain and Northern Ireland		Lowest 10%-18% of the country’s income
Singaporean		Lowest 10%-20% of the country’s income

#### 2.1.2 Implications for sustainable development

In 1987, the United Nations World Commission on Environment and Development defined the concept of sustainable development for the first time in its report Our Common Future, emphasizing the need to meet the development needs of current generations while safeguarding the interests of future generations. The concept emphasizes the balanced development of the economic, social, and environmental dimensions, laying the foundation for the subsequent multidimensional and hierarchical development of the theory of sustainable development [23]. The report clearly states that sustainable development aims to promote harmonious coexistence in the economic, social, and environmental spheres, providing an integrated framework for global development [24] This concept was inherited and deepened by the 2030 Agenda for Sustainable Development, released by the United Nations in 2015, which advocates a coherent and comprehensive strategy to achieve coordinated global development through the integration of three major areas [25].

#### 2.1.3 Relationship between poverty and sustainable development

As poverty research continues to advance, the focus of poverty research has gradually shifted from unidimensional poverty to multidimensional poverty, and several cutting-edge studies have endeavored to elaborate the link between "poverty eradication" and sustainable development, and there close relationship between poverty and sustainable development. On the one hand, poverty is an important cause of environmental degradation and resource depletion [26]. Poor groups are often overdependent on natural resources, leading to overexploitation of resources and ecological degradation [27]. On the other hand, environmental degradation exacerbates poverty, creating a vicious circle [28]. Hubacek et al. also validate the alignment of climate goals with poverty eradication from a global perspective [29]. Ruoqi Li et al. discuss the impacts and synergies of achieving different poverty eradication goals on air pollutants in China [30]. These novel papers provide insights into the integration of poverty reduction with economic, social, and environmental elements.

### 2.2 Coupled coordination related research

#### 2.2.1 Coupled field studies

In the context of the gradual transformation of research elements from one to many, the research on the coordinated development of multiple elements has gradually become the focus of scholars’ attention [31, 32], and the coupled coordinated relationship refers to the interactions and mutual influences between two or more systems that are connected in a certain way. The study of coupling is used to be widely used in empirical studies of the level of development of coupling between many systems, such as urbanization, industry, and transportation, at different scales and in different regions. Dong Liu reveals the spatial and temporal variability of the degree of match between agricultural water and soil resources by constructing a water-soil system, and explores the impact on food production [33]; The coupled coordination status of China’s water-environmental and socio-economic systems was analyzed, and a model of the coupled coordination degree of urbanization and ecological environment in Lianyungang was established by using the collected panel data [34]; Cui analyzed the coupling and coordination between the water environment system and the socio-economic system in China based on the coupling coordination degree model [35]; Empirical studies have been conducted to investigate the coupling relationship between tourism and the environment in Guilin City [36] and Silk Road Economic Belt [37]. These relevant studies have revealed interactions and the importance of context in a coupled tourism subsystem and environment subsystem; Zhang Shiwen studied the objective problems of economic development and environmental protection in the three northeastern provinces and proposed measures to improve regional economic development based on the coupling relationship [38]. Coupled coordination is gradually applied to various fields, which provides a direction for this paper to study sustainable poverty eradication strategies through the coupling of economy, nature, and society.

#### 2.2.2 Coupled measurement

At present, in the context of the gradual change of research elements from one to many, the study of the coordinated development of multiple elements has gradually become the focus of scholars. To study the coupling between multiple elements, a series of coupling evaluation models have been proposed, such as the system dynamics model [39], the planetary boundary theory [40], the EKC model [35], and the coupling coordination degree model (CCDM). The use of system dynamics modeling to systematically study social-ecological systems reflects the complexity of social-ecological systems to a certain extent [39]. However, the systems approach reflects the relationship between society and ecosystems only in terms of causality, and it is difficult to obtain quantitative results. Therefore, EKC and CCDM models have gradually emerged and been widely used. Compared with EKC, the coupled coordination degree model focuses more on describing the interactions between two subsystems and better reflects the degree of coordination between the elements in the system, therefore, many scholars have conducted relevant studies based on the coupled coordination degree model, for example, Li Yangfang established a coupled coordination degree model of urbanization and ecological environment in Lianyungang that using collected panel data [34]. Fang analyzed the coupling and coordination status of China’s water-environmental system and socio-economic system based on the coupling coordination degree model [35]. Overall, the above studies have achieved significant results in multi-factor coupling and coordination, which provides a good foundation for this study.

### 2.3 Governance of relative poverty

With the evolution of social and economic development, particularly following the eradication of absolute poverty according to current standards, relative poverty has progressively emerged as a societal issue, with its drawbacks becoming increasingly apparent. Consequently, addressing how to alleviate relative poverty and effectively manage it in the context of new development stages has become an urgent matter [41]. Numerous scholars have conducted extensive research on the governance of relative poverty. For instance, Based on some official statistics and survey data, Guan Xinping analyzes China’s current poverty problem and some important aspects of anti-poverty social policies and proposes social policies to reduce poverty by expanding the coverage of benefits raising the level of protection, increasing its public investment [42]. Cuong Viet Nguyen proposes a poverty reduction strategy from a policy perspective by comparing the poverty rate and the income and expenditure of independent households in provinces and districts as reported by the government [43], and Van Q. Tran proposes that enhancing the diversity of income sources of farming households can effectively reduce poverty by investigating the impact of income diversity on the transformation of poverty [44]. Fan Gang has proposed that addressing relative poverty should focus on five major challenges, including institutional safeguards, industrial cultivation, capacity building, humanistic development, and psychological services. Through the establishment of a multi-dimensional poverty indicator system, Wang Yanhui has developed targeted measures based on the identified factors for poverty management, combined with regional specificities [45]. Additionally, Zexian Gu has suggested a relative poverty pathway based on the analysis of poverty degree, type, and spatial distribution characteristics in 255 administrative villages in Nujiang Lisu Autonomous Prefecture, emphasizing rural revitalization, ecological environment management, eco-tourism, modern agriculture, mountainous agroforestry, and improvement of people’s livelihoods and well-being [46]. Most existing studies on regional relative poverty governance have qualitatively proposed relevant policy initiatives related to infrastructure, social security, and ecological environment, with some also employing quantitative methods to study poverty situations in specific regions, providing valuable insights for poverty eradication proposals.

### 2.4 Limitations in current research

The literature review discussed above has yielded numerous research findings on standards for measuring relative poverty, relative poverty characterization data, coupling, and governance countermeasures. However, there are still some limitations. In terms of the indicator system, there are more cases of adopting multi-dimensional thinking to measure relative poverty, but most of the data characterizing the indicator dimensions still use the traditional social census sampling data on an annual basis, which has a lag in identifying relative poverty, and at the same time, due to the limitations of the research data, the cost of acquiring the data is higher; although a few researches have also made use of remote sensing data for the identification of poverty-stricken areas, the accuracy of the identification is relatively low.In terms of relative poverty governance initiatives, fewer studies answer the question of where the internal subsystems of current poverty areas are about each other, without comprehensively considering the overall development of the region. In addition, the current governance initiatives on regional relative poverty are mainly based on qualitative research, supplemented by quantitative research, in which most of the quantitative research is based on the entire research sample, and the proposed initiatives on relative poverty governance are relatively broad and not comprehensive and targeted. Therefore, based on the theory of sustainable development and socio-economic-environmental composite ecosystem, we construct a multidimensional index system for regional relative poverty identification from the three dimensions of economy, society, and environment, integrating multi-source data such as crawlers, statistics, space, and other data related to the regional relative poverty with a faster update rate to determine the relative poverty areas through machine learning methods and adopt ArcGIS spatial analysis software, comprehensive evaluation model and Coupling coordination degree model, visualization of the current relative poverty in poverty and its internal subsystems overall coupling and coordination status, clarification of the type of poverty in each region, to achieve the overall coordinated and sustainable development of the regional economy-society-nature as the premise of targeted poverty eradication initiatives.

### 2.5 Research framework

**Fig 1:**
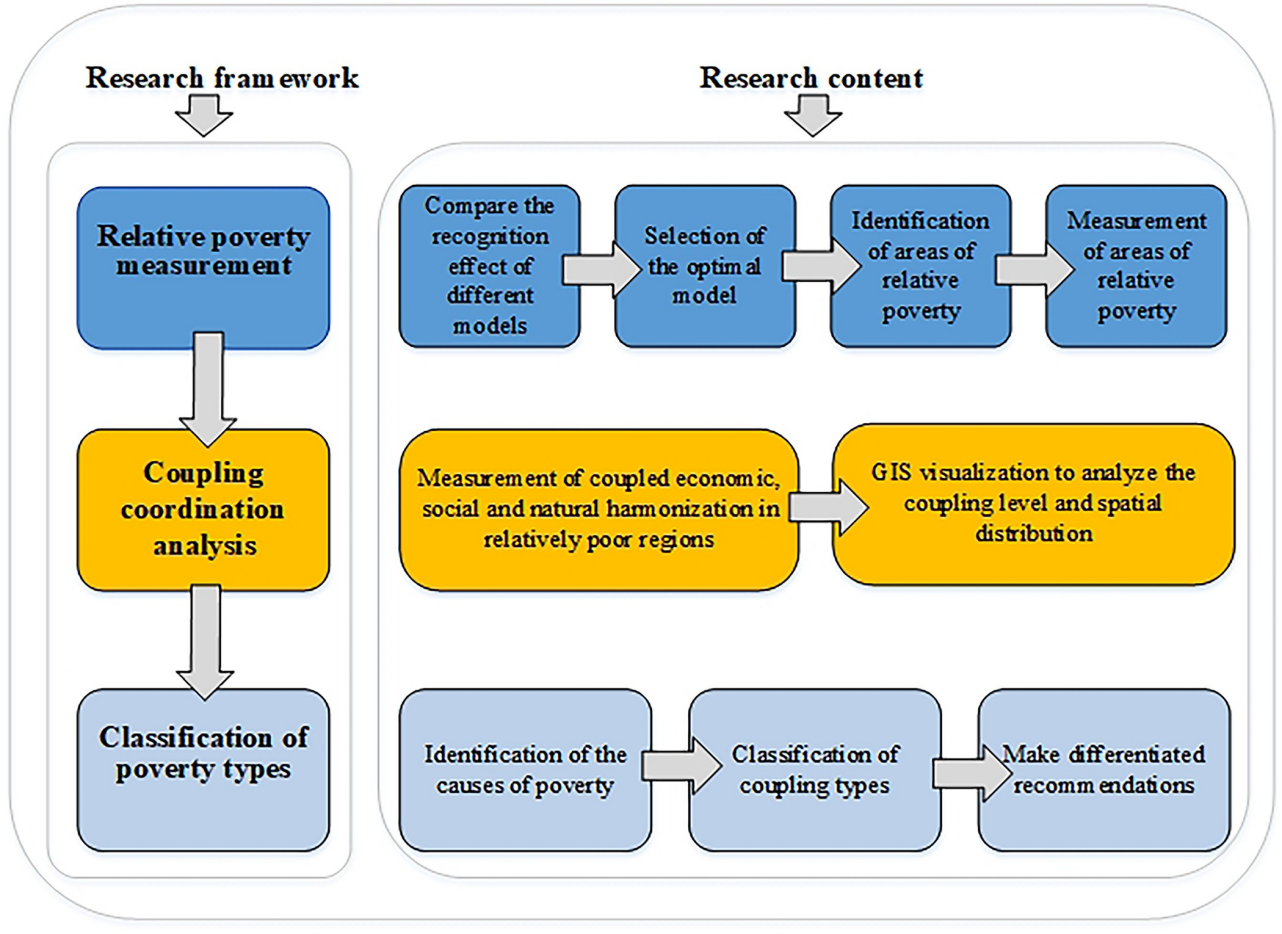
Research framework.

The research logic of "relative poverty measurement-coupled coordination analysis-poverty type classification" is followed. 1) Relative poverty measurement: by comparing the initial recognition effect of multiple machine learning models and the recognition performance of each model after parameter optimization, the optimal model is selected for the identification of relatively poor areas. The economic relative poverty index, social relative poverty index, environmental relative poverty index, and multidimensional relative poverty index of these areas were measured by using the comprehensive evaluation model, and the poverty degree and differences of relatively poor areas in China were analyzed. 2) Coupling coordination analysis: From the three dimensions of economy, society, and environment, the coupling coordination model was used to evaluate the degree of coupling coordination of these relatively poor areas, and the ArcGIS spatial analysis software was used to conduct visualization and analysis, to determine whether the "economy-society-nature" system of the current stage of each relatively poor area is in a coordinated state of development. 3) Classification of poverty types: To clarify the causes of poverty and the imbalance of internal system coupling in the current relatively poor areas, the poverty types and the poverty degree were classified into three dimensions: economic, social, and environmental. Finally, based on the poverty types of the current relatively poor areas and the results of their subsystem coupling and coordination, targeted poverty alleviation suggestions are put forward to provide a reference for alleviating the poverty situation of the current relatively poor areas in China. The specific research framework is shown in Fig 1.

## 3 Materials and methods

### 3.1 Study area

Based on the study of relative poverty from a regional perspective at the national level, the sample population is defined as 337 regions (prefectural cities, districts, autonomous prefectures, and leagues) covering 31 provinces (autonomous regions and municipalities directly under the central government) in the country based on the criteria of the national administrative division in 2022, excluding Taiwan Province of China, the Hong Kong Special Administrative Region (HKSAR) and the Macao Special Administrative Region (MSAR). In terms of time horizon, since the country is going to start shifting to relative poverty governance after 2020, the time horizon of the data was set as 2020–2022, taking into account the frequency of data updates.

### 3.2 Data source and processing

In terms of the relative poverty indicator system, the MPI is widely used, but the indicator system is more suitable for measuring the poverty level of micro subjects such as households or individuals and is less suitable for the study of regional poverty such as provinces, municipalities, and counties. We focus on the study of relative poverty sustainable development capacity, sustainable development theory, and socio-economic-environmental composite ecosystem both believe that economic activities, social public services, and the environmental environment develop and restrict each other. Therefore, this study adopts the socio-economic-environmental composite system framework to construct the indicator system.

Economic Dimension: The economic dimension includes the economic vitality, economic efficiency, and economic structure of the region: the index of house price is used to measure the economic vitality of the region [47], and GDP per capita is used to comprehensively measure the economic efficiency of the region [48], and the proportion of the value-added of the primary, secondary and tertiary industries to the gross domestic product is used to express the position and layout of the primary, secondary and tertiary industries in the total economy of the region and the economic structure of the region, respectively [49];Social Dimension: The social dimension includes infrastructure, social security and social influence. In terms of infrastructure, two indicators are selected to characterize the number of registered enterprises in the transportation, storage, and postal industry, and the number of registered enterprises in the information transmission, software, and information technology services industry [50]; in terms of social security, the number of educational institutions and the number of health and social work institutions per 10,000 people are selected to measure the level of local education development, medical and health care level, respectively, given the accessibility, comprehensiveness and frequency of updating of the data, and the number of resident population in each region. The number of educational institutions and the number of health and social work institutions per 10,000 people were chosen to measure the local education development level and health care level, respectively [51]. In terms of social influence, given that Baidu is a platform with a high market share in the domestic search engine market, the Baidu search index data was used to reflect the social influence of the region [51];Environmental dimensions: The environmental dimensions include climatic and topographic conditions, and the two major directions of climate and topography are chosen to characterize the natural environment of the region. In this paper, the temperature, rainfall, and air quality AQI are chosen to comprehensively reflect the climatic conditions of the region [52, 53], and the digital elevation DEM and average slope are chosen to reflect the topographic conditions of the region [54]. There are 3 dimensions, 8 directions, and 15 specific indicators, which are shown in Table 2. The selection of indicators in this study is scientific and representative and can measure the relative poverty of the region more comprehensively.

**Table 2:** Data sources for indicators.

Dimensions	Vectors	Indicators	Sources
Economic	Economic vitality	Housing price	Anjuman (https://www.anjuke.com/)
	Economic benefit	Per capita GDP	China Urban Statistics Progress Database and Guoxin Real Estate Information Network, National Economic and Social Development Statistics Bulletin, Statistical Yearbook, and official website announcements as supplementary channels
	Economic structure	The proportion of value added to primary industry	
		Proportion of added value of secondary industry	
		Proportion of value added of tertiary industry	
Social	Infrastructure	Number of registered enterprises in information transmission, software, and information technology services	Enterprise Search (https://www.qcc.com/)
		Number of registered enterprises in transportation, warehousing, and postal services	
	Social security	Number of health and social work facilities per 10,000 people	
		Number of educational institutions per 10,000 people	
	Social influence	Baidu search index	Baidu (https://index.baidu.com/v2/index.html#/)
Environmental	Climatic conditions	Air temperature	National Center for Environmental Information NCEI (https://www.ncei.noaa.gov/data/global-summary-of-the-day/archive/)
		rainfall	Data set of daily values of surface climate data in China
		Air Quality AQI	China Air Quality Monitoring Platform (https://www.aqistudy.cn/historydata/)
	Topographic condition	Digital elevation DEM	SRTM 90-meter DEM digital elevation database website (http://srtm.csi.cgiar.org/srtmdata/)
		Average grade	DEM extraction-based calculations (Derive Slope, ArcGIS)

Because the data for different indicators were collected at varying time scales, the data for temperature and rainfall were obtained daily, while the data for house prices and air quality AQI were obtained monthly. Other indicators were updated quarterly. To facilitate data integration later on, the daily temperature and rainfall data and the monthly house price and air quality AQI data for sample areas were uniformly converted into quarterly data. This involved summing up the daily data for temperature and rainfall for each quarter and dividing by the total number of days, as well as summing up the monthly data for house prices and air quality AQI for each quarter and dividing by the total number of months. Data with more than 50% missing values were removed, while those with fewer missing values were manually identified and supplemented using mean/median, nearest neighbor interpolation, or series data smoothing. Outliers were typically addressed by deletion, treating them as missing values, correcting them with the mean, or leaving them unprocessed. The preprocessing ultimately resulted in quarterly correlation data for each explanatory variable across 324 regions from Q1 2020 to Q4 2022,The processed data are shown in S1 Table.

### 3.3 Methods

#### 3.3.1 Relative poverty measurement

A multidimensional system of indicators was created to measure relative poverty everywhere from the perspective of economic, social, and environmental systems. It reflects the current level of poverty and spatial distribution patterns in these relative poverty areas using economic, social, environmental, and multidimensional relative poverty indices. Previous studies have commonly used the entropy value method and hierarchical analysis method to determine indicator weights, but these methods have limitations and can lead to errors in the results. This study, however, employs the SHAP importance value to quantitatively analyze the contribution weight of each indicator to regional relative poverty compared to other indicators within the corresponding dimension. By calculating the ratio of the SHAP importance value of each indicator variable to the importance value of all factors within its dimension, the weight of each indicator variable is obtained [55]. This approach takes into account the relative importance of each indicator factor to relative poverty compared to other indicators, resulting in scientifically and reasonably calculated weight values. The SHAP method, introduced by Lundberg and Lee in 2017, is used to explain the impact of input metrics on model output values and the level of importance of each metric in machine learning models.

In the calculation of indicator weights, the entropy value method and hierarchical analysis method are often used to determine the weights of indicators in previous studies, but these methods have certain defects and cause certain errors in the results. In this study, the SHAP importance value is used to quantitatively analyze the contribution weight of each indicator to regional relative poverty compared with other indicators under the corresponding dimension, i.e., by calculating the ratio of the SHAP importance value of each indicator variable to the importance value of all the factors under its dimension, the weight of each indicator variable is obtained [55]. This method comprehensively considers the relative importance of each indicator factor to relative poverty compared with other indicators, and the weight calculation results are scientific and reasonable.


$$\alpha (i)=\sum_{S\subseteq N\backslash \{i\}}\frac{|S|!(M-|S|-1)!}{M!}[v(S\cup \{i\})-v(S)]$$ (1)

Where N is the set of all indicators in the model; S is a subset of all indicators under multiple combinations of indicators; M is the number of indicators; *v*(*S*) is the prediction obtained by utilizing the set of indicators S; v(S∪{i}) is the prediction obtained by adding the indicator i on top of the set of indicators S; and $\frac{|S|!(M-|S|-1)!}{M!}$ denotes the weighting of the difference of the sample’s values based on the set of indicators S.The weights of the indicators are calculated as follows:

$$\begin{array}{l} w(x_{i})=\frac{\alpha (x_{i})}{\alpha (x_{1})+\alpha (x_{2})+\cdots +\alpha (x_{n})}=\frac{\alpha (x_{i})}{\sum_{i=1}^{n}\alpha (x_{i})}\\ w(y_{j})=\frac{\alpha (y_{j})}{\alpha (y_{1})+\alpha (y_{2})+\cdots +\alpha (y_{n})}=\frac{\alpha (y_{j})}{\sum_{j=1}^{n}\alpha (y_{j})}\\ w(z_{k})=\frac{\alpha (z_{k})}{\alpha (z_{1})+\alpha (z_{2})+\cdots +\alpha (z_{n})}=\frac{\alpha (z_{k})}{\sum_{k=1}^{n}\alpha (z_{k})} \end{array}$$ (2)

Among them, *w*(*x*_*i*_), *w*(*y*_*j*_), *w*(*z*_*k*_) are the weights of the indicator variables *x*_*i*_, *y*_*j*_, *z*_*k*_ under the economic, social, and environmental dimensions, respectively; *α*(*x*_*i*_), *α*(*y*_*j*_), *α*(*z*_*k*_) are the SHAP characteristic importance values of the indicator variables *x*_*i*_, *y*_*j*_, *z*_*k*_, respectively, the larger the value indicates that the importance of the variables *x*_*i*_, *y*_*j*_, *z*_*k*_ for the relative poverty status of the region is higher; $\sum_{i=1}^{n}\alpha (x_{i})$, $\sum_{i=1}^{n}\alpha (y_{j})$, $\sum_{i=1}^{n}\alpha (z_{k})$ are the sums of SHAP values of the indicator variables of the economic, social, and environmental dimensions, respectively, and n is the number of indicators under the respective dimensions. Several indicators.

The calculation of the weights of the dimensions is carried out based on the weights of the indicators, which are calculated as follows:

$$\begin{array}{l} w_{e}=\frac{\sum_{i=1}^{n}\alpha (x_{i})}{\sum_{i=1}^{n}\alpha (x_{i})+\sum_{j=1}^{n}\alpha (y_{j})+\sum_{k=1}^{n}\alpha (z_{k})}\\ w_{s}=\frac{\sum_{j=1}^{n}\alpha (y_{j})}{\sum_{i=1}^{n}\alpha (x_{i})+\sum_{j=1}^{n}\alpha (y_{j})+\sum_{k=1}^{n}\alpha (z_{k})}\\ w_{n}=\frac{\sum_{k=1}^{n}\alpha (z_{k})}{\sum_{i=1}^{n}\alpha (x_{i})+\sum_{j=1}^{n}\alpha (y_{j})+\sum_{k=1}^{n}\alpha (z_{k})} \end{array}$$ (3)

Where *w*_*e*_、*w*_*s*_、*w*_*n*_ represents the weights of the economic dimension, social dimension, and environmental dimension in the multidimensional relative poverty measurement index system respectively. To gain a better understanding of the poverty status in each relatively poor area across the three main aspects of economy, society, and environment, the economic, social, and environmental relative poverty indices for each region are computed using a multi-dimensional relative poverty measurement index system. The formula for calculating each relative poverty index is as follows:

$$\begin{array}{l} f(x)=\sum_{i=1}^{n}w(x_{i}){x_{i}}^{'}\\ g(y)=\sum_{j=1}^{n}w(y_{j}){y_{j}}^{'}\\ h(z)=\sum_{k=1}^{n}w(z_{k}){z_{k}}^{'} \end{array}$$ (4)

Among them, *f*(*x*)、*g*(*y*)、*g*(*z*) denotes the economic relative poverty index, social relative poverty index, and environmental relative poverty index, respectively; *w*(*x*_*i*_), *w*(*y*_*j*_), and *w*(*z*_*k*_) are the weights of the indicator variables *x*_*i*_, *y*_*j*_, and *z*_*k*_ under the economic, social, and environmental dimensions, respectively; and ${x_{i}}^{'}$, ${y_{j}}^{'}$, and ${z_{k}}^{'}$ are the standardized values of the indicators under the economic, social, and environmental dimensions, respectively, and the standardization of the indicators is processed by the method of extreme deviation.

Furthermore, to gain a more thorough comprehension of the overall poverty status in each specific area, the multidimensional poverty index for the region is computed by building upon the calculation of the one-dimensional poverty index. This involves using the combined weights of the dimensions within the poverty measurement system to assess the dimensions and then deriving the multidimensional poverty index for each region using the provided formulas.


$$RPI=f(x)w_{e}+g(y)w_{s}+h(z)w_{n}$$ (5)

Where *RPI* represents the multidimensional relative poverty index; *f*(*x*)、*g*(*y*)、*h*(*z*) represents the economic relative poverty index, the social relative poverty index, and the environmental relative poverty index, respectively; and *w*_*e*_、*w*_*s*_、*w*_*n*_ represents the combined weights of the economic dimension, the social dimension, and the environmental dimension, respectively.

#### 3.3.2 Coupling coordination analysis

Coupling refers to the interaction and interdependence between two or more systems or forms of movement, involving mutual coordination, promotion, and influence on each other [56] Coupling indicates the level of interaction within the system or its internal components [57]. The more interconnected the systems are, the greater their level of interaction and reliance on each other. To ensure stable development between the systems, it is important to achieve a balanced level of interaction, leading to the introduction of the concept of coordination to assess the quality of interaction between the systems. Coordination involves establishing a positive relationship between two or more systems or their internal components, enabling the overall system to develop in a mutually beneficial and harmonious manner [58]. The level of coupling coordination is utilized to assess the level of compatibility and smooth progress among the components of the system or within the system.

The equation for determining the degree of coupling in economic-social-environmental systems is as follows:

$$C={\left\{\frac{u_{1}\times u_{2}\times u_{3}}{{\left[(u_{1}+u_{2}+u_{3})/3\right]}^{3}}\right\}}^{1/3}$$ (6)

In Eq (6), C is the degree of economic-social-environmental system coupling, the larger its value indicates that the interaction among economic, social and environmental systems in the relatively poor areas is stronger, and its value range is [0, 1]; *u*_1_, *u*_2_, *u*_3_ are the rankings of the economic poverty index, social poverty index, and environmental poverty index of each relatively poor area after standardization, respectively; the larger the *u*_1_, *u*_2_, *u*_3_ is, the greater is the extent of the region’s economic poverty, the extent of the social poverty, the lower is the extent of environmental poverty, and the greater is the extent of the region’s economic poverty, The larger the index, the lower the degree of economic poverty, social poverty and environmental poverty in the region. To determine the type of coupling degree belonging to each relatively poor region, combined with the basis of coupling degree in many previous studies, the coupling degree is divided into four categories [59, 60]. To judge the type of coupling degree to which each relatively poor region belongs, combined with the basis for the division of coupling degree in many previous researches, the coupling degree is divided into four types, as shown in Table 3.

**Table 3:** Coupling degree types of economy-society-environmental system.

Number	Coupling (physics)	Type of coupling
1	0≤C≤0.3	low-level coupling
2	0.3<C≤0.5	antagonistic coupling
3	0.5<C≤0.8	break-in coupling
4	0.8<C≤1	High-level coupling

The degree of coupling can only judge the degree of interaction between the subsystems or internal elements of the system, but it cannot judge whether the system is in a state of orderly coordinated development or disordered chaotic development. Therefore, to judge the overall level of coordinated development of the system and then introduce the coupling coordination degree model, which integrates the economic-social-environmental coupling status C and the development level T, the specific calculation formula is as follows:

$$D=\sqrt{C\times T}$$ (7)

$$T=\alpha \times u_{1}+\beta \times u_{2}+\delta \times u_{3}$$ (8)

Where D represents the degree of coordination of the coupled economic-social-environmental system, measuring the level of coordinated development of the three; T is the comprehensive evaluation index of the economic-social-environmental system of the relatively poor areas; *α*、*β*、*δ* are the pending coefficients of the economic, social, and environmental systems, respectively; the pending coefficients of *α*、*β*、*δ* are proposed to be 0.4, 0.2, and 0.4, drawing on relevant research and combining with the distribution of the relatively poor areas [61, 62]. The coefficients to be determined are 0.4, 0.2, and 0.4. To identify the types of coupling coordination belonging to different relatively poor areas, combined with the division basis of many previous studies, the degree of coupling coordination is divided into four types [63, 64], and the specific division criteria are shown in Table 4

**Table 4:** Coupling coordination types of economy-society-environmental system.

Number	Degree of coupling coordination	Type of coupled coordination
1	0≤D<0.4	recessionary dysregulation
2	0.4≤D<0.5	Protracted disorder
3	0.5≤D<0.6	Barely coordinated developmental
4	0.6≤D≤1	Harmonized developmental

#### 3.3.3 Classification of poverty types

Based on the economic relative poverty index, social relative poverty index, and environmental relative poverty index of the national sample areas calculated in the previous section, calculate the mean value of the whole sample under each dimension, and compare the relative poverty index of each dimension of the relatively poor areas with the mean value of the corresponding dimension poverty index of the sample areas, and designate the relatively poor area as poor under the dimension if it is larger than the mean value, and clarify the poverty situation of each relatively poor area under the economic, social, and environmental dimensions. Economic, social, and environmental dimensions [65]. The calculation method is as follows:

$$\begin{array}{l} C_{f}=P_{f}^{i}-\sum_{t=1}^{N}f(x)/N\\ C_{g}=P_{g}^{i}-\sum_{t=1}^{N}g(y)/N\\ C_{h}=P_{h}^{i}-\sum_{t=1}^{N}h(z)/N \end{array}$$ (9)

Eq (9), *C*_*f*_、*C*_*g*_、*C*_*h*_ is the difference between the relative poverty index of the relatively poor areas under the economic, social, and environmental dimensions respectively, and the average value of the relative poverty index of all the 324 sample areas in the country under the dimension; $P_{f}^{i}、P_{g}^{i}、P_{h}^{i}$ is the economic relative poverty index, the social relative poverty index, and the environmental relative poverty index of the relatively poor area I; $\sum_{t=1}^{N}f(x)/N、\sum_{t=1}^{N}g(y)/N、\sum_{t=1}^{N}h(z)/N$ is the average value of the economic relative poverty index, the average value of the social relative poverty index, and the average value of the environmental relative poverty index of all the 324 sample areas in the country respectively. Are the average values of the economic relative poverty index, social relative poverty index, and environmental relative poverty index of 324 sample areas in China? If *C*_*f*_、*C*_*g*_、*C*_*h*_≤0, it means that the relatively poor region is not poor in economic, social, and environmental dimensions respectively, and if *C*_*f*_、*C*_*g*_、*C*_*h*_>0, it means that the relatively poor region is poor in economic, social, and environmental dimensions respectively.

## 4. Results

### 4.1 Relative poverty measurement

#### 4.1.1 Identification of regions of relative poverty

In recent years, advanced information technologies like big data and machine learning have started to be gradually utilized in poverty management, offering a dependable and efficient technical method for accurately identifying impoverished regions and populations. The task of identifying relative poverty in different regions can be seen as a binary classification issue in machine learning. This study selects four models for classification problems—logistic regression, support vector machine (SVM), random forest, and XGBoost—and compares their accuracy, precision, recall, F1-score, and AUC value in the ROC curve. The findings indicate that XGBoost is the most effective under default parameters and is ultimately chosen as the recognition model. Tables 5 and 6 present the specific values of each evaluation index before and after parameter tuning.

**Table 5:** Comparative analysis of the initial recognition effect of models.

Mold	Accuracy	Precision	Recall	F1-Score	ROC_AUC
XGBoost	0.914	0.832	0.747	0.787	0.853
random forest	0.907	0.873	0.658	0.750	0.816
SVM	0.742	0.447	0.897	0.597	0.799
logistic regression	0.824	0.662	0.350	0.458	0.651

**Table 6:** Comparative analysis of recognition effect after model tuning.

Mold	Accuracy	Precision	Recall	F1-Score	ROC_AUC
XGBoost	0.891	0.809	0.637	0.713	0.798
random forest	0.911	0.883	0.671	0.763	0.817
SVM	0.809	0.538	0.719	0.616	0.776
logistic regression	0.833	0.648	0.466	0.542	0.699

**Fig 2:**
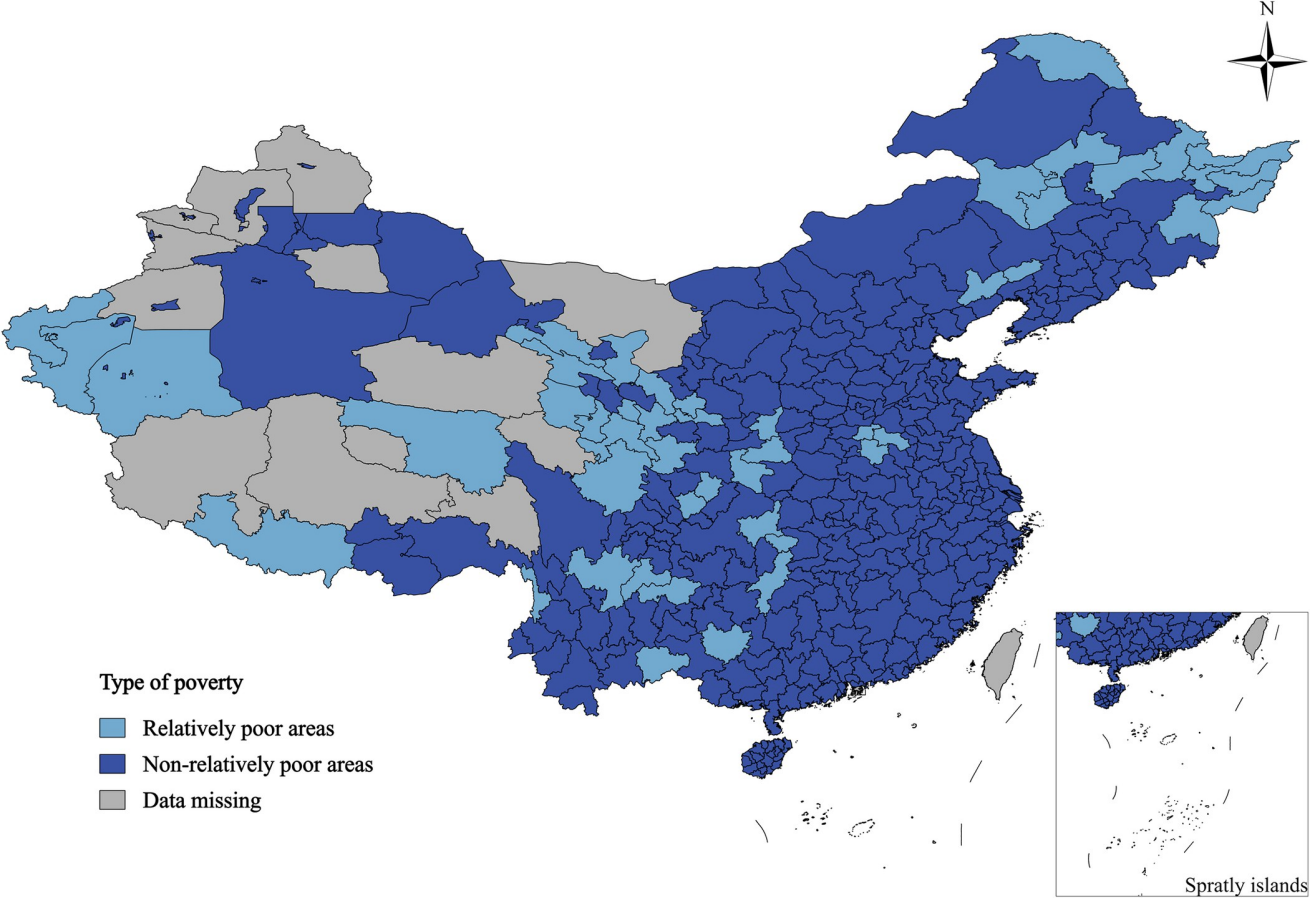
Relatively poor areas of the country by the end of 2022.

According to the XGBoost model, a total of 48 regions were identified as being in a state of relative poverty by the end of 2022 (Fig 2), spread across the country. These areas generally exhibit a spatial distribution pattern characterized by "large dispersion, small aggregation". The regions with relative poverty are primarily concentrated in the northwest and northeast of China, including Gansu, Qinghai, Tibet, Xinjiang, Heilongjiang, and other areas where relative poverty is more severe. In Sichuan, Yunnan, and other provinces, relative poverty areas are mainly concentrated in autonomous regions and remote cities, such as Aba Tibetan and Qiang Autonomous Prefecture, Liangshan Yi Autonomous Prefecture, Nujiang Lisu Autonomous Prefecture, Wenshan Zhuang and Miao Autonomous Prefecture, and others. Additionally, relative poverty areas in the northeast regions of Inner Mongolia, Heilongjiang, Jilin, Liaoning, etc. are mainly found in areas such as Daxing’anling, Qiqihar, Jixi, Hegang, Fuxin, Baicheng, and others. Furthermore, some areas in Henan Province, Hubei Province, Hunan Province, Shaanxi Province, and other provinces are also identified as being in a state of relative poverty, including Kaifeng City, Shangqiu City, Zhoukou City, Enshi Tujia and Miao Autonomous Prefecture, Huaihua City, and others. Non-relatively poor areas in China are mainly concentrated in the Yangtze River Delta region, located in the southeastern part of the country. These areas, due to their advantageous geographical location and high level of industrial development, exhibit weaker levels of poverty compared to the rest of the country. This identification aligns with the actual overall development level of China’s current regions and further confirms the accuracy, scientificity, and rationality of the XGBoost model used in this study to identify the relatively poor areas.

#### 4.1.2 Measurement of relative economic-social-environmental poverty

Before examining the level of coordination within the interconnected "economic-social-environmental" system in economically disadvantaged regions, a comprehensive evaluation model is used to calculate the economic, social, environmental, and multidimensional relative poverty indices of these areas. The scores of these poverty indices are then combined to better understand the degree and variations of poverty in these regions in China. Subsequently, the coupling and coordination of the three systems (economy, society, and nature) are analyzed based on their poverty index scores to determine whether they are in a state of coordinated development in these economically disadvantaged regions. The results of this analysis will serve as a reference for future poverty eradication measures.

Based on the multidimensional index system for the identification of relative poverty, after calculating and organizing the weights of each index and dimension, the multidimensional index system for the measurement of relative poverty and the weights of economic, social, and environmental dimensions are finally obtained. The specific index weights are shown in Table 7.

**Table 7:** Multidimensional system of relative poverty indicators.

Dimensions	Dimension weights	Vectors	Indicators	Indicator weights
Economic	0.5867	Economic vitality	Housing price	0.2312
		Economic benefit	Per capita GDP	0.2485
		Economic structure	The proportion of value added to primary industry	0.3838
			Proportion of added value of secondary industry	0.1112
			Proportion of value added of tertiary industry	0.0253
Social	0.2059	Infrastructure	Number of registered enterprises in information transmission, software, and information technology services	0.2522
			Number of registered enterprises in transportation, warehousing, and postal services	0.3835
		Social security	Number of health and social work facilities per 10,000 people	0.1198
			Number of educational institutions per 10,000 people	0.1496
		Social influence	Baidu search index	0.0949
Environmental	0.2074	Climatic conditions	Air temperature	0.0616
			rainfall	0.0601
			Air Quality AQI	0.1315
		Topographic condition	Digital elevation DEM	0.4716
			Average grade	0.2752

**Fig 3:**
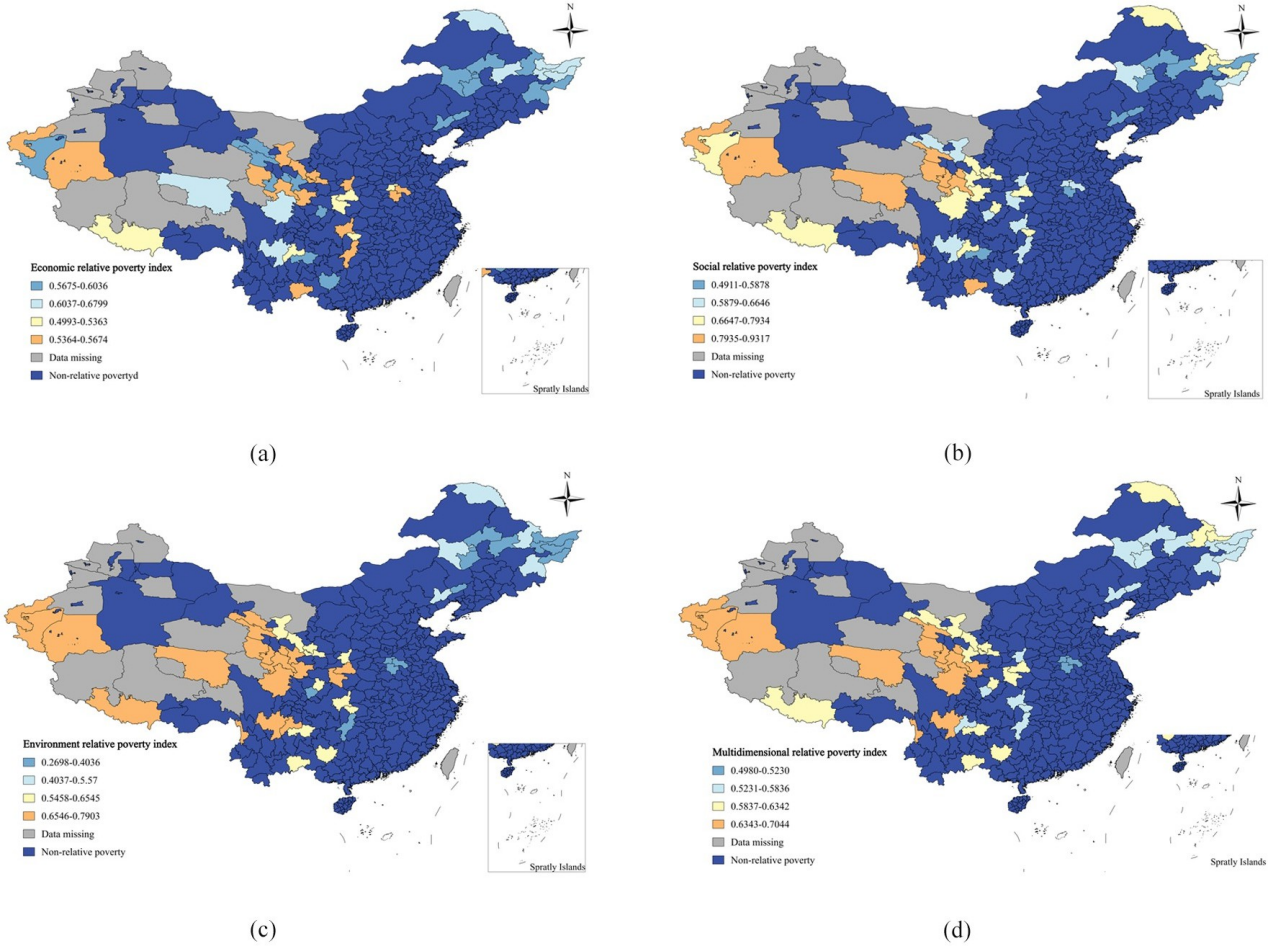
Spatial distribution of relative poverty levels. (a) Economic relative poverty index,(b) Social relative poverty index,(c)Environmental relative poverty index,(d)Multidimensional relative poverty index.

By combining the scores of economic, social, environmental, and multidimensional relative poverty indices, the spatial distribution pattern of relative poverty indices reveals that areas with high economic relative poverty indices are situated in Heilongjiang and Sichuan Provinces. Meanwhile, regions with significant levels of social relative poverty are predominantly found in the northwestern region, particularly in the autonomous regions of Yunnan, Gansu, Qinghai, and Xinjiang Provinces. The areas with severe levels of environmental relative poverty are still concentrated in the northwest of China, such as Gansu, Qinghai, Sichuan Tibetan, Xinjiang, and other regions. The multi-dimensional relative poverty index is higher in regions mainly in Yunnan, Guizhou, Sichuan, Xinjiang, Gansu, Qinghai, and other areas, which are typical of China’s deeply impoverished regions. Future poverty management efforts in China should continue to focus on these deeply impoverished areas, addressing economic, social, environmental, and other multi-dimensional aspects of poverty alleviation comprehensively. In comparison to these deeply impoverished areas, when implementing poverty alleviation measures in regions with lower multidimensional relative poverty indexes, such as Henan and Heilongjiang, targeted and efficient poverty alleviation efforts can be carried out based on their specific weaknesses. The relevant results are presented in Fig 3

### 4.2 Coupling coordination analysis

**Fig 4:**
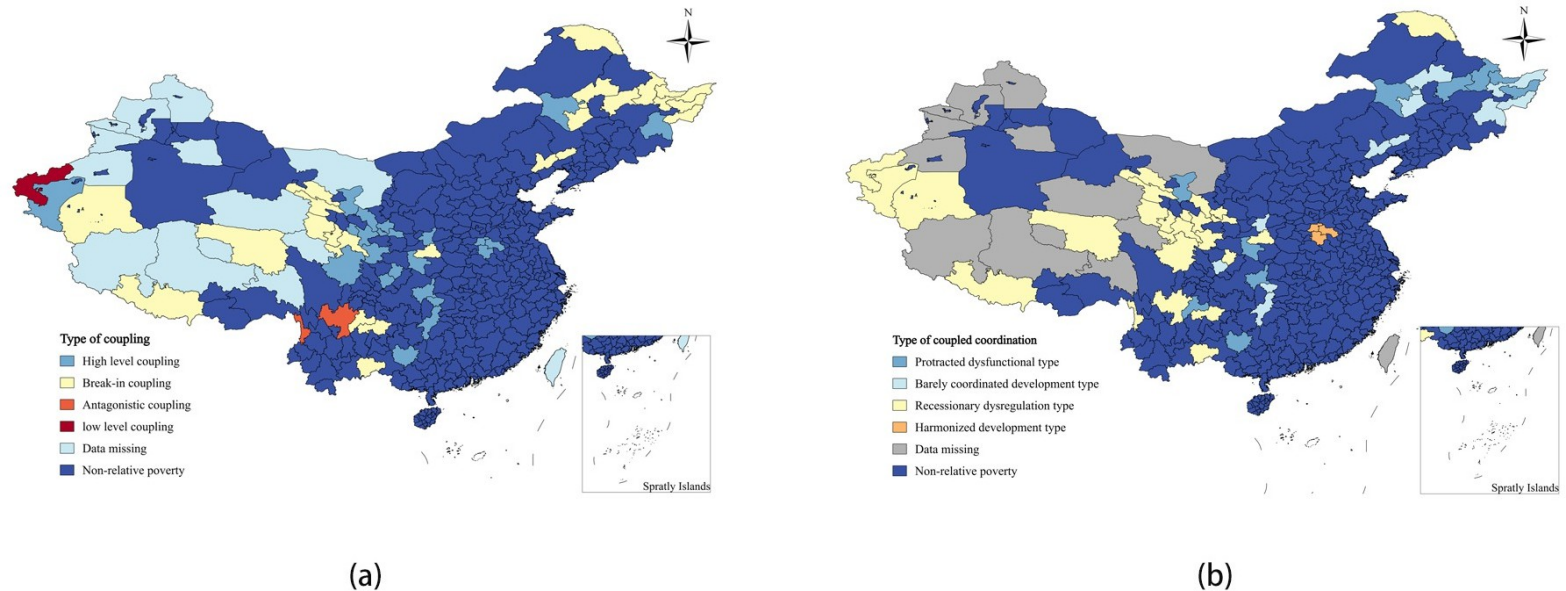
Relative poverty area coupling and types of coupling coordination. (a)Type of coupling,(b)Type of coupling coordination.

The region’s relative poverty is influenced by its economic base, social welfare and security, and lack of environmental resources. These factors collectively form the regional economic-social-environmental system. Internally, the economic base, social welfare and security, and environmental environment subsystems interact and mutually affect each other. It is crucial to analyze this interaction and influence, as well as to coordinate the relationship between the systems, to effectively manage relative poverty sustainably. To assess the association among economic, social, and environmental subsystems, the coupling degree model is utilized to calculate the coupling degree of the current multidimensional relative poverty system in each relatively poor region. However, this model cannot differentiate whether the subsystems are in a beneficial coordinated development or a harmful interaction. Therefore, it is essential to combine the coupling coordination degree model to measure the coupling coordination degree of the economic-social-environmental system in each relatively poor region. The results of the spatial distribution map are displayed in Fig 4.

#### 4.2.1 Analysis of coupling degree

The average coupling degree of the current multidimensional relative poverty system in each relatively poor region is 0.7496, indicating a high overall level of coupling. The correlation among the economic, social, and environmental systems in these regions is also high, with strong interaction among the various sub-systems. Most relatively poor areas show relatively strong correlation and interaction among their economic, social, and environmental subsystems. Regions with the highest coupling scores, such as Baiyin, Dingxi, and Guyuan in Shaanxi, Gansu, Henan, Hunan, and Sichuan, exhibit the strongest poverty interactions across economic, social, and environmental dimensions. Conversely, Kizilsu Kirghiz Autonomous Prefecture shows a low level of coupling, with greater relative poverty in the social and environmental dimensions compared to the economic dimension, leading to variability and asynchrony in relative poverty across these dimensions. Nujiang Lisu Autonomous Prefecture and Liangshan Yi Autonomous Prefecture are in antagonistic coupling, with differences in poverty levels across each dimension. Nujiang Prefecture experiences greater topographical variation and has seen economic improvement in recent years due to national policies, while Liangshan Yi Autonomous Prefecture has higher economic and environmental relative poverty compared to the social dimension, resulting in an overall state of antagonistic coupling.

#### 4.2.2 Analysis of the coupling coordination degree

The average coupling coordination degree of the current relatively poor areas is 0.3978, indicating a low overall level of coordination in the economy-society-nature relationship, leading to disorder. Most subsystems in these areas do not support each other and coexist harmoniously but rather impose mutual constraints and experience disordered development, which significantly hinders overall coordinated development. In the future, targeted governance measures should be taken to address these dysfunctional areas. Coupled coordinated regions are mainly in the central part of the Northeast, with fewer constraints from environmental conditions. On the other hand, coupled dysfunctional regions are mainly clustered in Xinjiang, Qinghai, Gansu, Heilongjiang, Yunnan, Guizhou, Sichuan, and other regions, mostly due to poor environmental environment, imperfect infrastructure, and various social security facilities. To manage these relatively poor regions, it is essential to understand the interactions between economic, social, and environmental subsystems within each region, aiming for comprehensive development and improvement of the environmental environment, social infrastructure, and welfare protection. This approach will optimize the regional industrial layout and drive economic and social development in all aspects, ultimately achieving sustainable and coordinated regional development.

### 4.3 Classification of poverty types

Currently, the economic, environmental, and social systems in many of the less affluent areas are not in balance, and the reasons for their relative poverty or the interconnection of these systems are unclear. The formation of regional poverty is the result of multiple factors, and efforts to address it should be comprehensive. Identifying the specific type of poverty in each region is crucial for targeted poverty eradication proposals. Therefore, we initially assess the relative poverty status of each region in terms of economy, society, and nature. Then, using ArcGIS spatial analysis software, we categorize the relatively poor regions into severe, moderate, and mild poverty based on environmental breakpoints.

#### 4.3.1 Relative economic deprivation

**Fig 5:**
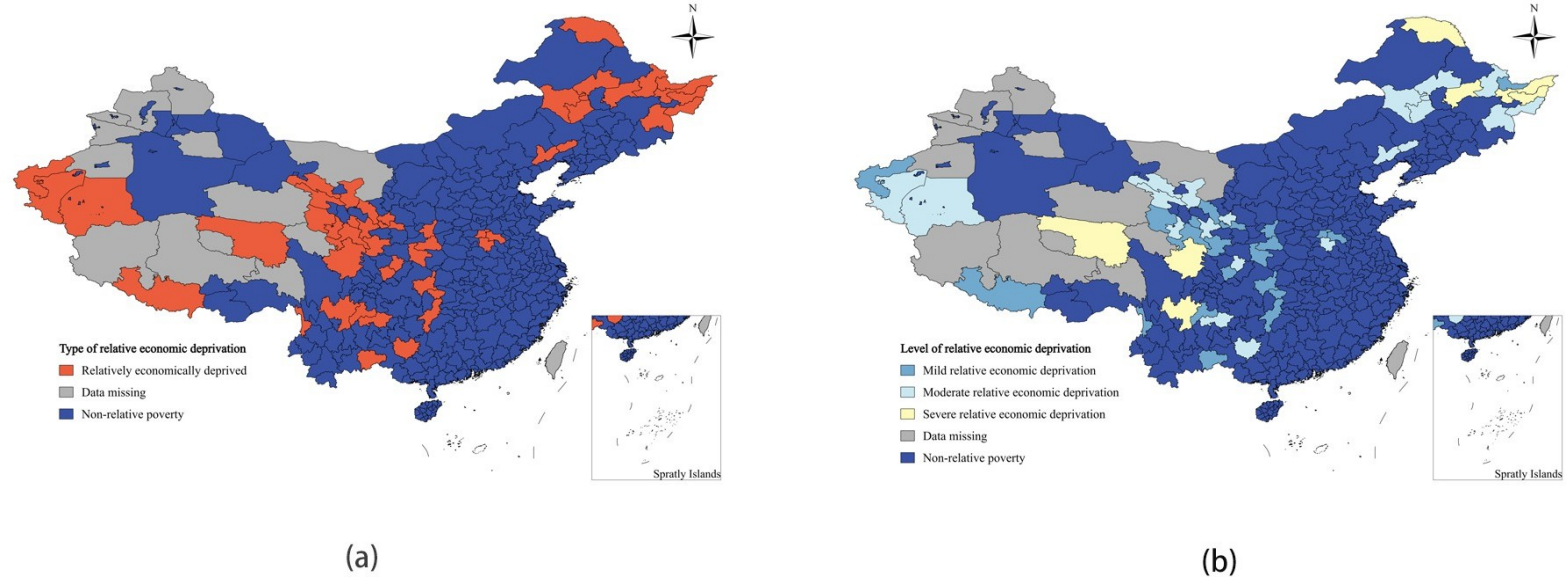
Spatial distribution map of economically relatively poor areas and poverty levels. (a) Type of relative economic deprivation,(b) Level of relative economic deprivation.

The average relative economic poverty index for the 324 sample areas nationwide was 0.4926. It was observed that all areas classified as relatively poor were also economically disadvantaged. In terms of economic relative poverty division, the largest number of areas fell into the moderate economic relative poverty category, totaling 21 and accounting for 43.75% of the total. Light and severe economic relative poverty areas made up 39.58% and 16.67% respectively, indicating that the current economic poverty level in these areas is moderate to low. The heavy economic relative poverty areas were mainly concentrated in Heilongjiang Province, Sichuan Province, and Qinghai Province, such as Hegang City, Shuangyashan City, Daxinganling District, and Liangshan Yi Autonomous Prefecture (Fig 5, Table 8). These areas rely mainly on the primary industry, with less developed secondary and tertiary industries, resulting in relatively low regional economic development and infrastructure lag. The moderately economically impoverished areas were mainly located in Qinghai Tibet, Gansu Province, Heilongjiang Province, Liaoning Province, and other areas, such as Qiqihaer, Jixi, Wuwei, and Haibei Tibetan Autonomous Prefecture. These areas have slightly better economic development compared to heavily impoverished areas but still face challenges due to factors such as geographic location and environmental environment. Mild relative economic poverty was mainly found in the central and southern regions, such as Shangqiu, Ankang, Huaihua, Baiyin, and Hainan Tibetan Prefecture, which have favorable geographic locations and regional resources that have not been fully utilized.

**Table 8:** Statistics of spatial relative poverty in various regions in China.

Dimensions	Overall relative poverty index	Different levels of relative poverty as a proportion of relatively poor areas		
		mildly	moderately	severe
Relative economic deprivation	0.4926	39.58%	43.75%	16.67%
Relative social deprivation	0.5859	25%	35.42%	20.83%
environmental relative poverty	0.4570	14.58%	22.92%	39.58%

#### 4.3.2 Relative social deprivation

**Fig 6:**
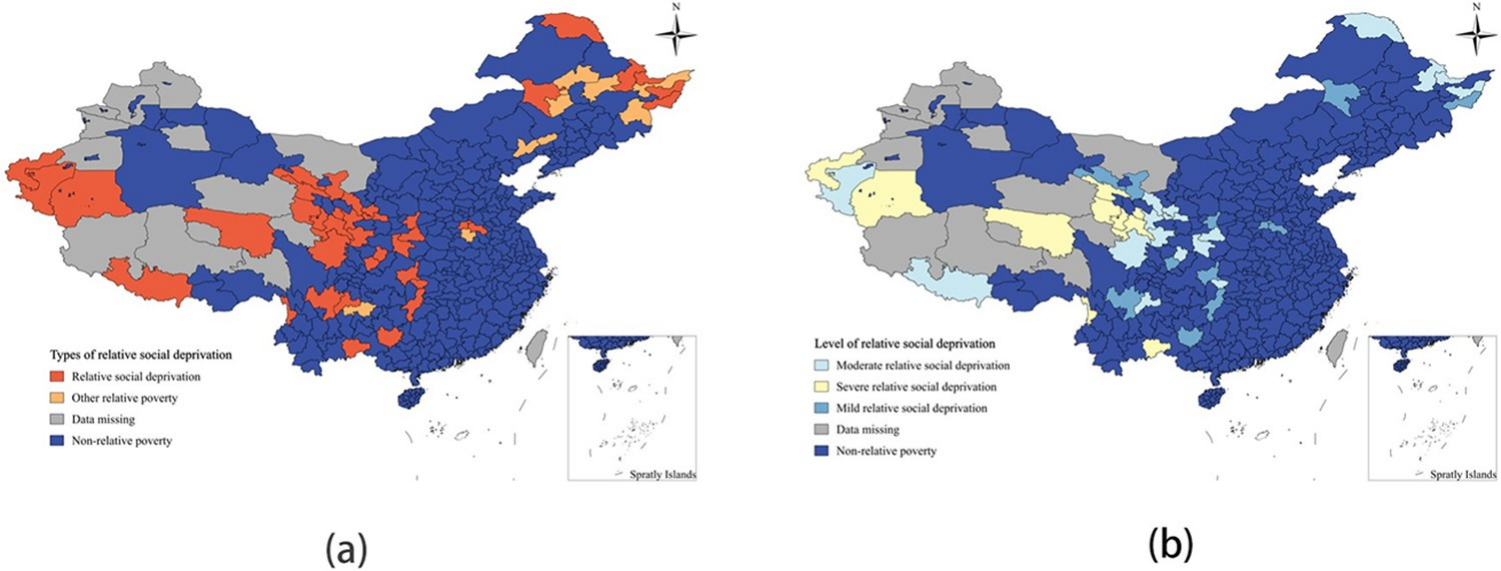
Spatial distribution map of socially relatively poor areas and poverty levels. (a)Type of relative social deprivation,(b) Level of relative social deprivation.

The average social relative poverty index value for the 324 sample regions across the country is 0.5859. Out of the total number of relatively poor regions, 39 regions, or 81.25%, are characterized by relative poverty under the social dimension. In terms of social relative poverty classification, the largest number of areas falls under moderate social relative poverty, with 17 areas accounting for 35.42% of the total. The second largest category is mild social relative poverty, with 12 areas making up 25% of the total. Severe social relative poverty areas total 10, accounting for 20.83% of the total. Severe social relative poverty is mainly concentrated in the northwestern regions of Yunnan, Gansu, Qinghai, and Xinjiang, particularly in autonomous prefectures such as the Nujiang Lisu Autonomous Prefecture, the Linxia Hui Autonomous Prefecture, the Haibei Tibetan Autonomous Prefecture, and the Hotan region (Fig 6 and Table 8). These regions face challenges such as high transportation and information infrastructure costs due to their closed geographical location and poor environmental environment, leading to lower infrastructure development compared to the national average. This lack of infrastructure restricts the flow of material, capital, and technology, exacerbating the poverty in these regions. Mildly socially relatively poor areas are widespread in provinces and regions such as Henan, Hubei, Hunan, Sichuan, and Gansu, where the overall level of infrastructure and public service guarantees is relatively lower. Non-social relative poverty areas include Fuxin City, Chaoyang City, Baicheng City, Qiqihar City, Jiamusi City, Mudanjiang City, Suihua City, Zhoukou City, and Bijie City. Fuxin City, for example, is a major transportation hub in the western part of Liaoning Province, with a well-connected transportation network that promotes connectivity with the outside world. Baicheng City, located at the junction of the three northeastern provinces, has a well-developed transportation system and inland waterway trade with Russia. Qiqihar City has been increasing investment in the transportation industry, improving rural roads, and enhancing rail and aviation capacity, while also improving social security services such as education and healthcare for residents.

#### 4.3.3 Relative environmental deprivation

**Fig 7:**
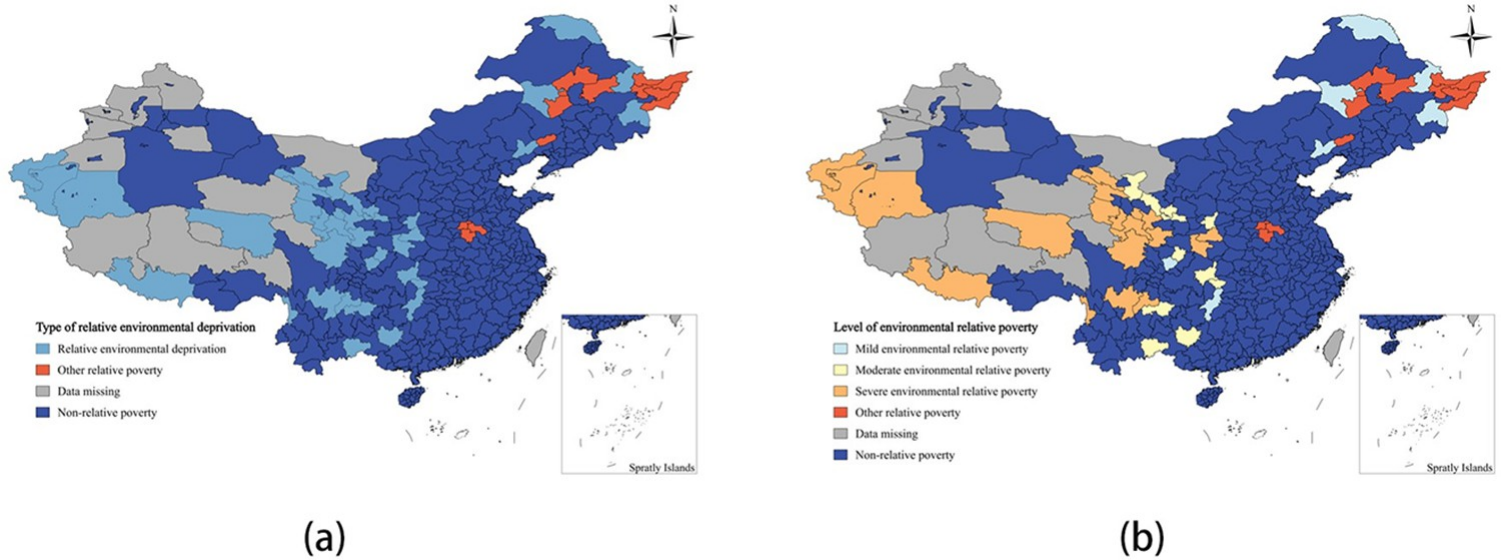
Spatial distribution map of environmental relatively poor areas and poverty levels. (a) Type of relative environmental deprivation,(b) Level of relative environmental deprivation.

The average environmental relative poverty index value for the 324 sample areas is 0.4570. Out of these, 37 areas are relatively poor in the environmental dimension, making up 77.08% of the total number of relatively poor areas. The severe environmental relative poverty areas are the most numerous, totaling 19 and accounting for 39.58% of the total number of relative poverty areas. There are 7 mild environmental relative poverty areas, making up 14.58% of the total, and 11 moderate environmental relative poverty areas, accounting for 22.92% of the total (Fig 7). The areas with environmental relative poverty are mainly concentrated in the northwestern part of the country, including Yunnan, Guizhou, Sichuan, and part of the northeastern region. Severe environmental relative poverty is mainly found in the southern Border States such as Gansu, Qinghai, and Yunnan, which are characterized by alpine and arid regions with fragile ecological environments, high altitudes, and frequent environmental disasters. Medium environmental relative poverty areas are more scattered, including regions like Hubei, Hunan, Yunnan, Guizhou, Sichuan, Gansu, and Shaanxi, which have undulating topography and weak industrial and agricultural development. Mild environmental relative poverty areas are mainly in mountainous terrains such as Huaihua City, Xing’anmeng, Chaoyang City, and Daxing’anling. Non-environmental relative poverty areas are mainly in the central and northeastern regions, characterized by plains, suitable temperatures, and richer ecological resources compared to other relatively poor areas.

### 4.4 Differentiated poverty eradication strategy

**Fig 8:**
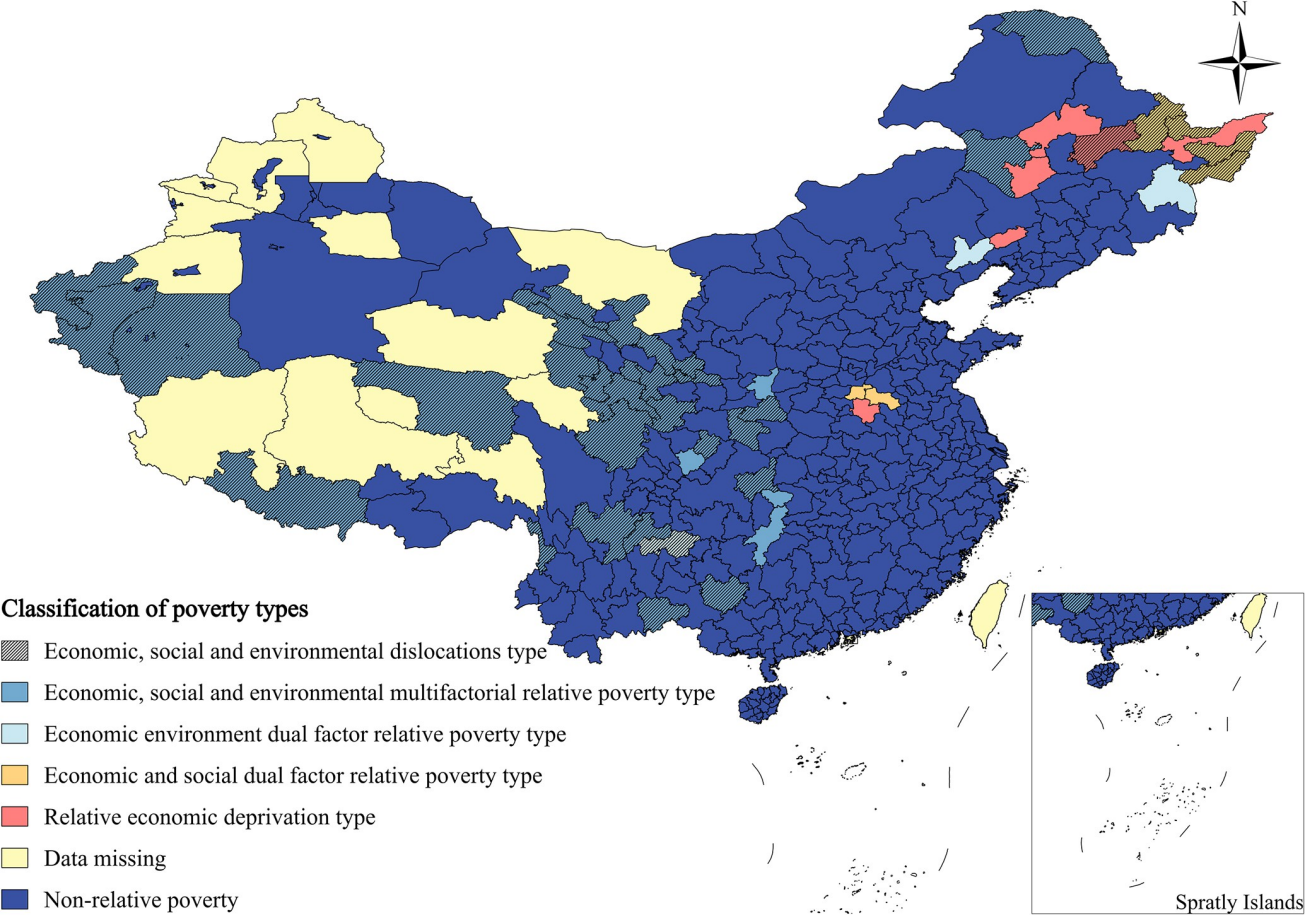
Spatial distribution of poverty types in relatively poor areas.

The aforementioned analysis has identified three dimensions of relative poverty: economic, social, and environmental. Each dimension has been elucidated about its impact on poverty. Based on this categorization, relative poverty areas have been classified into seven types: economic, social, environmental, economic-social two-factor, economic-environmental two-factor, social-environmental two-factor, and economic-social-environmental multi-factor. However, no areas fall into the social, environmental, and social-environmental types, resulting in a final classification of four poverty types. To offer targeted and effective suggestions for poverty alleviation, the results of the economic-social-environmental system coordination for each relatively poor area and its respective poverty type have been amalgamated. The spatial distribution pattern is depicted in Fig 8. The analysis indicates that there are six regions (12.5%) with economic relative poverty, nine regions (18.75%) with two-factor poverty, and 33 regions (68.75%) with multi-factor poverty. The majority of relative poverty areas exhibit poverty in all three dimensions, suggesting that relative poverty is a comprehensive multifactorial issue. Consequently, poverty governance should be comprehensive and multifaceted. Unidimensional relative poverty areas allow for a clearer understanding of the poverty mechanism and more precise poverty alleviation efforts. However, for dual-factor or multi-factor relative poverty, a more complex approach is required, combining various aspects for localized and targeted treatment. In terms of spatial distribution, economic-social-environmental multi-factor relative poverty is predominantly located in the northwest, southwest, and part of the northeast, representing typical poverty-stricken areas in China. Notably, 87.88% of these areas are in a state of dysfunctionality, indicating that the economic, social, and environmental subsystems are mutually constraining and exacerbating poverty in these regions.

#### (1) Relative economic deprivation

There are six districts categorized as unidirectional relative economic deprivation, mainly located in the north-east. These districts have favorable socio-environmental conditions but are relatively lagging in economic development. Among them, the dysfunction of the subsystem within Suihua is because its level of social and environmental development is higher than its economic dimension. This economic backwardness seriously hinders the overall development of society and nature, resulting in systemic dysfunction. The coupling between subsystems within the remaining five regions is in a state of harmonization, indicating that the economy, society, and nature coexist harmoniously and promote each other. However, the level of economic development in these regions is still lower than the national average, resulting in relative poverty at the economic level. In solving the problem of poverty management in relatively economically poor regions, future poverty alleviation efforts should build on existing social and environmental development and take advantage of their favorable geographic location and environmental setting to increase regional economic incomes. This can be achieved through financial support and the enhancement of the region’s capacity for autonomous development. Strategies such as developing and utilizing local resources, focusing on manpower, capital, and technology, and developing special industrial projects should be adopted to overcome economic shortcomings and accelerate industrial development. Optimizing the industrial structure and establishing an industry-related benefit linkage mechanism with local characteristics can enhance the comprehensive strength of the region and promote sustainable economic development. In addition, while vigorously developing the regional economy, the supporting role of infrastructure and the environmental environment must be strengthened to ensure the sustainable development of the region.

#### (2) Economic and social dual factor relative poverty type

There are six economic-social two-factor relative poverty areas, mainly concentrated in Henan and Heilongjiang, which have generally better environmental and ecological environments, but slow socio-economic development due to a variety of factors. Compared with Jixi, Kaifeng, and Shangqiu, Hegang, Shuangshanshan, and Yichun are in a state of dislocation, indicating that the economic and social development of these three regions lags behind the level of local environmental resources, and lagging in the economic and social dimensions has led to their overall dislocation, which makes it even more urgent for these regions to strengthen the input and development of these two dimensions. And make full use of their good ecological resources to alleviate socio-economic problems. These regions should increase their economic and social inputs and development, make full use of their good ecological resources, alleviate socio-economic poverty, and enable the whole system to achieve coordinated development. When poverty alleviation work is carried out in such regions, local advantageous resources should be combined to vigorously develop eco-tourism, specialty industries, and so on. In addition, a sound infrastructure is an important guarantee for achieving poverty alleviation and high-quality development in the region, and it is necessary to strengthen the construction of regional transportation, information, logistics, and other infrastructure to lay the foundation for high-quality development of the industry. At the same time, it is necessary to strengthen education for poverty alleviation and health, increase regional education, medical care, and other social security efforts, rational allocation of education and medical resources, solve some of the region’s difficulties in schooling, medical care, and other livelihood issues, and promote the equalization of basic public services and a high level of equalization. It should be noted that, for this type of region, when carrying out economic and social poverty alleviation, it is necessary to fully protect the local ecological environment, and not to sacrifice the ecological environment as the price of economic development. In this regard, local governments can establish an environmental protection system with the government, the public, enterprises, and social organizations as the main body and mutual supervision to maintain the regional ecological environment and guarantee the sustainability of regional development, while achieving more income generation. Sustainability of regional development.

#### (3) Economic and environmental dual factor relative poverty type

Areas categorized as relatively poor due to a combination of economic and environmental factors include Chaoyang City, Mudanjiang City, and Bijie City. In Bijie City, for example, the overall economic-social-environmental system is dysfunctional, and both economic and environmental poverty are important constraints to social development. In contrast, Chaoyang and Mudanjiang have low to medium levels of economic and environmental development compared to the rest of the country, hindering regional development and leading to relative poverty. Effective governance of these areas requires taking advantage of location, infrastructure, and social aspects, addressing the shortcomings of the original economic structure, adjusting the industrial structure, promoting investment, enhancing corporate social responsibility, mobilizing local resources, and vigorously developing secondary and tertiary industries. Implement a development model led by the government and involving leading enterprises, cooperatives, and the poor. In terms of the environmental environment, cooperative afforestation of barren mountains and returning farmland to forests can absorb the poor population, paying attention to the restoration and maintenance of vegetation and preventing deaths caused by negligent maintenance. Strengthening ecological and environmental remediation and water conservancy construction, combined with local resource advantages, will promote industrial upgrading and the development of specialty industries. This includes selecting vegetation with economic value for ecological construction projects, such as forest herbs and specialty aquaculture, to realize economic gains and protect the ecological environment.

#### (4) Economic, social, and environmental multifactorial relative poverty type

Thirty-three regions in China are categorized as having a multifactorial relative poverty typology, including economic, social, and environmental aspects. These regions are mainly located in the northwest, Yunnan, Guizhou, Sichuan, and parts of the northeast, and are characterized by comprehensive poverty. Among them, 29 regions, including Xing’anmeng, Daxing’anling region, and Enshi Tujia and Miao Autonomous Prefecture, have a dysfunctional coupling of economic, social, and environmental systems, accounting for 87.88% of the total. This indicates a general trend of malignant development in these regions. Addressing poverty in these areas requires an integrated approach that takes into account the interplay of economic, social, and environmental aspects to promote coordinated development. Efforts to reduce poverty in these areas should include restructuring industries, integrating sustainable economic development into regional strategies, and integrating industrial development with local environmental resource capacities. In addition, areas with complex terrain and poor infrastructure can utilize biological, mineral, and cultural resources to develop industries such as ecotourism. Areas affected by factors such as slope and elevation, such as the three southern prefectures of Xinjiang and the city of Shigatse, can develop medium-sized agriculture and explore the ecological cycle of combining agriculture and animal husbandry. For ecologically fragile areas prone to geological disasters, measures such as relocation and resettlement and centralized resettlement will be taken, and ecological construction projects such as afforestation and soil erosion control will be implemented. Increasing the level of self-sufficiency in the region is crucial to eradicating relative poverty. This includes strengthening infrastructure, education, health care, and public services, as well as enhancing information networks, transportation facilities, and support for education and health care. These measures are aimed at increasing the self-reliance, self-development, and risk-resistance of the inhabitants of these areas, ultimately breaking the bonds of geographical poverty and realizing sustainable economic and social development in harmony with the environment.

### 4.5 Discussion

The identification results of this paper show that the relative poverty areas are mainly concentrated in the northwest and northeast of China, such as Gansu, Qinghai, Tibet, Xinjiang, Heilongjiang, and other areas, which is in line with the results of Fan Jie et al [66]. and the calculation of the relative poverty level using the multi-dimensional relative poverty index shows that the areas with higher relative poverty are mainly distributed in the areas of Yunnan, Guizhou, Sichuan, Xinjiang, Gansu, Qinghai and other areas, all of which are typical of China’s deep poverty zones, which is consistent with Cheng Fu’s research results [67]. China’s municipal relative poverty shows a ladder-like distribution from east to west, and the relative poverty areas are distributed all over the country, generally showing a spatial distribution pattern of "large dispersion, small aggregation", which is in line with the reality of unbalanced and inadequate regional development in China. The above results reflect the reliability of the research results to a certain extent.

However, we analyze the coupling and coordination among economy, society, and environment based on previous studies, clarify the current stage of the subsystems within economy, society, and environment, and the interaction among subsystems, and find that 70.83% of the relatively poor areas are in a state of dysfunction in terms of the degree of coupling and coordination and that the areas with dysfunctional coupling in terms of spatial distribution are concentrated in regions such as the three southern border regions, Qinghai, Gansu, Yunnan, Sichuan and other regions. The spatial distribution of the coupled dysfunctional areas is concentrated in the three southern border regions, Qinghai, Gansu, Yunnan, Sichuan, and other regions. The above findings provide policymakers with important references for accurately identifying problems, optimizing resource allocation, adopting comprehensive management measures, promoting cross-regional cooperation, dynamic monitoring and evaluation, and scientific decision-making support, which can help improve the efficiency and effectiveness of poverty alleviation.

## 5 Conclusions, managerial implications and limitations

### 5.1 Conclusions

This study offers a comprehensive understanding of relative poverty at the municipal level in China and provides targeted poverty alleviation strategies that align with the Sustainable Development Goals (SDGs). The innovative XGBoost model, developed through data integration, significantly enhances the accuracy and timeliness of poverty identification, achieving an impressive accuracy rate of 91.4%, surpassing traditional methods. Furthermore, the study’s analysis of coupling and coordination reveals complex relationships between the economic, social, and environmental subsystems in poverty-stricken areas, with 70.83% of these systems identified as dysfunctional, primarily in the southern regions of Xinjiang, Qinghai, Gansu, Yunnan, and Sichuan. This insight highlights the need for precise intervention measures. The classification of poverty types into four categories—relative economic deprivation, economic and social dual factor relative poverty, economic and environmental dual factor relative poverty, and economic, social, and environmental multifactorial relative poverty—highlights the spatial variability of poverty characteristics and facilitates the development of targeted regional poverty reduction strategies. The unique contribution of this study lies in its advanced data-driven approach, its nuanced understanding of the complexities of poverty, and its strategic recommendations for more effective poverty alleviation efforts.

### 5.2 Managerial implications

Based on the findings of the study, we now make the following specific recommendations: (1) For policymakers the findings of the study can help them understand the multidimensional nature of relative poverty and its interactions with economic, social, and environmental systems. This will help them to design and implement more comprehensive and targeted pro-poor policies, especially in determining resource allocation and formulating long-term development plans. (2) For local governments and community managers who are responsible for implementing poverty alleviation programs, the methodology for identifying regional relative poverty and the analysis of the degree of coupled coordination provided by the study can guide them to locate poverty areas more precisely, optimize the allocation of resources for poverty alleviation, and monitor the effectiveness of policy implementation. (3) For NGOs and social enterprises, the classification of different poverty types and the analysis of coupling coordination can provide a basis for intervention and help them design more effective community support and development programs to promote the sustainable development of the local economy and the enhancement of social well-being.

Through these management insights, the relevant beneficiaries can better understand and respond to the problem of relative poverty and take more scientific and effective measures to realize the coordinated development of the socio-economic environment and the sustainable governance of poverty.

### 5.3 Limitations and future research

Despite the theoretical and practical significance of this study, there are still certain limitations that suggest possible avenues for future research. (1) In terms of the research object, this study takes 337 regions across the country as the research object to research relative poverty governance, which has both expanded the scope of regional research and narrowed the scale of the research object compared with most of the previous studies. It fails to conduct more detailed and in-depth research at a more micro scale, such as county and village areas. (2) In terms of poverty eradication countermeasures for relatively poor regions, this study only proposes targeted poverty eradication countermeasures for each region, taking into account the type of poverty and the coupling situation. The specific factors affecting poverty in each region have not been taken into account, and in subsequent studies, poverty eradication countermeasures should be narrowed down from the dimensional level to the level of the specific factors affecting poverty, so that more targeted poverty alleviation measures can be proposed.

## Supporting information

S1 Table Post-processed data. (XLSX)
